# Forebrain‐Specific Transgene Rescue of 11β‐HSD1 Associates with Impaired Spatial Memory and Reduced Hippocampal Brain‐Derived Neurotrophic Factor mRNA Levels in Aged 11β‐HSD1 Deficient Mice

**DOI:** 10.1111/jne.12447

**Published:** 2017-01-09

**Authors:** S. Caughey, A. P. Harris, J. R. Seckl, M. C. Holmes, J. L. W. Yau

**Affiliations:** ^1^ UoE/BHF Centre for Cardiovascular Science University of Edinburgh Edinburgh UK; ^2^ Centre for Cognitive Ageing and Cognitive Epidemiology University of Edinburgh Edinburgh UK

**Keywords:** ageing, hippocampus, corticosterone, watermaze, BDNF

## Abstract

Mice lacking the intracellular glucocorticoid‐regenerating enzyme 11β‐hydroxysteroid dehydrogenase type 1 (11β‐HSD1) are protected from age‐related spatial memory deficits. 11β‐HSD1 is expressed predominantly in the brain, liver and adipose tissue. Reduced glucocorticoid levels in the brain in the absence of 11β‐HSD1 may underlie the improved memory in aged 11β‐HSD1 deficient mice. However, the improved glucose tolerance, insulin sensitisation and cardioprotective lipid profile associated with reduced peripheral glucocorticoid regeneration may potentially contribute to the cognitive phenotype of aged 11β‐HSD1 deficient mice. In the present study, transgenic mice with forebrain‐specific overexpression of 11β‐HSD1 (Tg) were intercrossed with global 11β‐HSD1 knockout mice (HSD1KO) to examine the influence of forebrain and peripheral 11β‐HSD1 activity on spatial memory in aged mice. Transgene‐mediated delivery of 11β‐HSD1 to the hippocampus and cortex of aged HSD1KO mice reversed the improved spatial memory retention in the Y‐maze but not spatial learning in the watermaze. Brain‐derived neurotrophic factor (BDNF) mRNA levels in the hippocampus of aged HSD1KO mice were increased compared to aged wild‐type mice. Rescue of forebrain 11β‐HSD1 reduced BDNF mRNA in aged HSD1KO mice to levels comparable to aged wild‐type mice. These findings indicate that 11β‐HSD1 regenerated glucocorticoids in the forebrain and decreased levels of BDNF mRNA in the hippocampus play a role in spatial memory deficits in aged wild‐type mice, although 11β‐HSD1 activity in peripheral tissues may also contribute to spatial learning impairments in aged mice.

Chronically elevated circulating glucocorticoids (GC; cortisol in humans and corticosterone in rodents) as a consequence of impaired hypothalamic‐pituitary‐adrenal (HPA) negative‐feedback regulation often accompany ageing and cognitive decline 1, 2, 3. Prolonged exposure of the brain to stress levels of GCs is considered to contribute to the cognitive deficits in aged individuals 4, 5. Indeed, animal studies have shown that chronic stress or high GC levels cause hippocampal dendritic atrophy, impaired synaptic plasticity, decreased neurogenesis and impaired memory 6, 7. Maintenance of low corticosterone (CORT) levels throughout life in rats prevents age‐associated changes in hippocampal morphology and impaired cognition, supporting high CORT levels as a cause rather than a consequence of hippocampal damage 8.

Glucocorticoids bind to two types of intracellular corticosteroid receptors in brain, the mineralocorticoid receptor (MR) and the glucocorticoid receptor (GR), to modulate learning and memory 9. Prior to receptor binding, GC levels can be modulated by the action of 11β‐hydroxysteroid dehydrogenases, which interconvert active and inactive GCs. 11β‐Hydroxysteroid dehydrogenase type 1 (11β‐HSD1)1 is highly expressed in the liver, adipose tissue and brain where it acts predominantly as a 11β‐reductase *in vivo* to regenerate active GCs (cortisol, corticosterone) within cells from inert 11‐keto forms (cortisone, 11‐dehydrocorticosterone), thus effectively amplifying local GC levels 10. The other isozyme 11β‐HSD2, acts as an exclusive 11β‐dehydrogenase inactivating GCs and is mostly expressed in the distal nephron, thus protecting otherwise nonselective MRs from GCs *in vivo* 11. In the adult brain, 11β‐HSD1 is the sole isozyme expressed in regions important for cognition such as the hippocampus and cortex 10.

11β‐HSD1 generated GCs play a crucial role in hippocampus‐dependent memory deficits associated with ageing 12. Aged mice with a life‐long deficiency of 11β‐HSD1, despite having elevated plasma CORT levels similar to those of aged wild type mice, exhibit lower intra‐hippocampal CORT levels and a spatial memory performance similar to that of young adult mice 13.

11β‐HSD1 deficient mice also exhibit a protective metabolic phenotype, as indicated by reduced glucose and insulin levels when on high‐fat diets 14, 15. Increased glucose levels over time are associated with cognitive decline in nondiabetic nondemented elderly subjects 16. Furthermore, animal models of hyperglycaemia exhibit impaired spatial learning 17, 18. It is therefore possible that the deficiency of 11β‐HSD1 in peripheral tissues may contribute to the improved cognitive phenotype of aged 11β‐HSD1 deficient mice. If the cognitive effects are solely a consequence of a lack of 11β‐HSD1 in brain, then we predict that forebrain specific transgene rescue of 11β‐HSD1 would reverse the improved cognition and any associated changes in hippocampal mRNA expression in aged 11β‐HSD1 deficient mice. To test this, we inter‐crossed transgenic forebrain‐specific 11β‐HSD1 overexpression mice 19 with global 11β‐HSD1‐deficient mice 14. In addition to MR and GR, we examined brain‐derived neurotrophic factor (BDNF) mRNA levels in the hippocampus given its important role in regulating synaptic plasticity mechanisms that underlie learning and memory 20 and its modulation by GCs 21.

## Materials and methods

### Animals

Transgenic mice with forebrain‐specific overexpression of 11β‐HSD1 under the CamIIK promoter were generated as described previously 19. Mice hemizygous for the transgene [referred to as transgenic (Tg) mice] were intercrossed with global 11β‐HSD1 deficient mice (*hsd11b1* ^*−/−*^; referred to as HSD1KO) 22 to generate *hsd11b1* ^*−/−*^ mice with forebrain‐specific expression of *hsd11b1* (referred to as Tg+HSD1KO) and without the transgene (referred to as HSD1KO). Both Tg and *hsd11b1* ^*−/−*^ mice were congenic on C57BL/6J strain. Nontransgenic [wild‐type (WT)] littermates were used as *hsd11b1* ^*+/+*^ controls. Male mice were used in the studies and maintained under standard conditions (12 : 12 h light/dark cycle, lights on 07.00 h, *ad lib*. access to standard chow and water) until experimentation. All animal procedures were approved by the University of Edinburgh Ethical Review committee and were carried out to the highest standards under the UK Animals Scientific Procedures Act, 1986 and the European Communities Council Directive of 24 November 1986 (86/609/EEC).

### Cognitive behavioural tests (Y‐maze and watermaze)

Basal tail venesection blood samples were taken from all mice between 08.30 h and 10.30 h 1 week prior to behavioural testing. Aged (24 months) HSD1KO, Tg+HSD1KO and WT mice were first tested in the Y‐maze to assess their spatial recognition memory retention as described previously 13. The Y‐maze apparatus consisted of three enclosed Plexiglas arms (length 50 cm, width 11 cm, height 10 cm) surrounded by visible extra maze spatial cues. On the day of Y‐maze testing, all mice were transported to the behavioural testing room in their home cages and left to acclimatise to the environment for 30 min. A handful of bedding material from each cage was added to the Y‐maze and mixed evenly to distribute the combined scents of the mice covering the floor of the maze. During trial 1, each mouse was allowed to freely explore two arms of the Y‐maze with one arm blocked (‘novel’ arm) for 5 min before returning to their home cage. Following a 1‐min inter‐trial interval (ITI), each mouse was returned to the Y‐maze with all arms now open for another 5‐min exploration. The time spent in the novel arm was calculated as a percentage of the total time in all three arms. The 1‐min ITI test ensured that the aged mice were engaging in the novelty of the task and had no visual problems seeing the spatial cues around the maze. All mice were re‐tested in the Y‐maze (with new cues around maze) 3 days later with a 2‐h ITI to measure spatial memory. The mice movements in the arms of the maze were tracked and analysed using anymaze software (Stoelting, Dublin, Ireland).

At least 1 week after Y‐maze testing, aged mice were trained in the watermaze as described previously 13 to measure spatial learning and memory. Young 6‐month‐old WT mice were included to confirm impaired spatial memory with ageing in *hsd11b1* ^*+/+*^ controls. Mice were first given 3 days of nonspatial training (four 90‐s trials per day with curtains around the pool to obscure external cues) to find the submerged platform marked with a visible plastic building brick (Lego A/S, Billund, Denmark) protruding on top. This tests for visual, motivational or motor deficits that might influence their performance in the spatial learning task. Mice unable to find the platform within 20 s on day 3 were excluded. For spatial training, mice were given 20 trials (20‐min ITI) over 5 consecutive days with no curtains around pool so they could navigate towards the hidden platform using the spatial cues around the room. Each trial started with the mouse facing the wall of pool at one of four randomly chosen locations and ended when they found the platform or when 90s had elapsed, in which case they were guided to the platform by hand. After 30s on the platform, the mice were returned to their home cage. Twenty‐four hours after the last training trial, each mouse was given a probe test, where they were placed in the pool (with platform removed) and left to swim freely for 60s. Swim paths were tracked using a video camera mounted in the ceiling and data analysis was conducted using watermaze software (Actimetrics, Evanston, IL, USA). Twenty‐four hours later, mice were weighed and culled by cervical dislocation. The brains were removed with one half hemisphere frozen on powdered dry ice for *in situ* hybridisation and the remaining half hemisphere was dissected with the hippocampus and cortex, frozen on dry ice and stored at −80 °C. Livers were collected, frozen on dry ice and stored at −80 °C, whereas the adrenals were stored in formalin at 4 °C. Excess fat was dissected from each adrenal prior to weighing on a microbalance.

### 11β‐HSD1 enzyme activity

Liver, cortex and hippocampal samples were homogenised in 500 μl of homogenising buffer [5 mm Tris, 1 mm ethylenediaminetetraacetic acid (EDTA), 20% glycerol] and assayed for 11β‐dehydrogenase activity as described previously 23. Samples were diluted in C buffer (10% glycerol, 50 mm sodium acetate, 1 mm EDTA; pH 6.0) to give a 50 μg/ml protein concentration for liver, a 200 μg/ml protein concentration for the cortex and a 400 μg/ml protein concentration for the hippocampus. The assay, containing 10 mm 3H‐corticosterone as a tracer and 2 mm NADP as a co‐factor, was incubated at 37 °C for 20 min for liver samples, and 4 h for hippocampus and cortex samples. The steroids were extracted with ethyl acetate and the percentage conversion of [^3^H]‐corticosterone to [^3^H]‐11‐dehydrocorticosterone was analysed by thin‐layer chromatography.

### Corticosterone radioimmunoassay

Plasma corticosterone levels were determined using an in‐house radioimmunoassay as described previously 24 with corticosterone antiserum kindly donated by Dr C. Kenyon (University of Edinburgh, Edinburgh, UK).

### *In situ* hybridisation

Antisense RNA probes for MR and GR were generated by *in vitro* transcription from plasmid vectors containing the appropriate cDNA insert, in the presence of [^35^S]‐UTP with SP6‐ and T7‐RNA polymerases, respectively, as described previously 25. For the detection of BDNF mRNA, mouse brain cDNA was used to generate ‘floppy’ polymerase chain reaction (PCR) template for probe synthesis. Primers (see Supporting information, Table S1) with 5′ extensions containing phage polymerase consensus sites were used to generate the BDNF cDNA template corresponding to bases 843–1362 of the mouse BDNF mRNA (GenBank accession number AY057908) 26. cDNA was pooled from several reactions and purified with High Pure PCR product Purification Kit (Roche, Burgess Hill, UK). Nucleotide sequencing revealed 100% homology between the amplied fragment and previously reported sequence. Antisense and sense cRNA probes (520 bp length) were prepared from the cDNA template by *in vitro* transcription in the presence of [^35^S]‐UTP (Perkin Elmer, Waltham, MA, USA) using T3 or T7 RNA polymerase, respectively.

Cryostat (10 μm) coronal brain sections at the level of the dorsal hippocampus from the behaviourally tested animals thaw‐mounted onto microscope slides were hybridised with the [^35^S]‐UTP labelled cRNA antisense probes for MR, GR and BDNF essentially as described previously 25. The specificity of the BDNF antisense cRNA probe was first tested prior to the main experiment in control brain sections along with the sense BDNF cRNA probe. Slides were dipped in photographic emulsion (NTB‐2; Kodak, Rochester, NY, USA) and exposed at 4 °C for 20 days before being developed (Kodak D19) followed by counterstaining with pyronin (1%). Silver grains were counted within a fixed circular area over individual cells under a × 40 light microscope objective using the KS300 image analysis system (Carl Zeiss, Oberkochen, Germany). The analysis was carried out blind to genotype, 15–18 cells/subregion were assessed for each animal and background (determined over areas of white matter) subtracted.

### Statistical analysis

Y‐maze data were analysed by two‐way anova with percentage time in the novel arm (1‐min ITI and 2‐h ITI) and genotype as the independent variables. Within animal comparisons of the percentage time in arms of the Y‐maze were analysed by multiple paired t‐tests with a Bonferroni adjusted P < 0.025. Watermaze data (visible platform and spatial learning) were analysed by two‐way repeated measures anova with escape latency over days of training and genotype as the independent variables. Probe test data were analysed by two‐way anova with percentage time in the different quadrants and genotype as independent variables. *In situ* hybridisation mRNA data were analysed by two‐way anova with subregions and genotype or age as independent variables. Enzyme bioactivity, plasma corticosterone and adrenal weight data were analysed using one‐way anova for the effects of genotype. All data were analysed using prism, version 6 (GraphPad Software Inc., San Diego, CA, USA) and statistical differences were determined using post‐hoc Bonferroni's multiple comparisons test (adjusted P‐values are shown where appropriate). All data are presented as the mean ± SEM.

## Results

### Rescue of forebrain 11β‐HSD1 activity in 11β‐HSD1‐deficient mice by CamIIK‐HSD1 transgene

**Figure 1:**
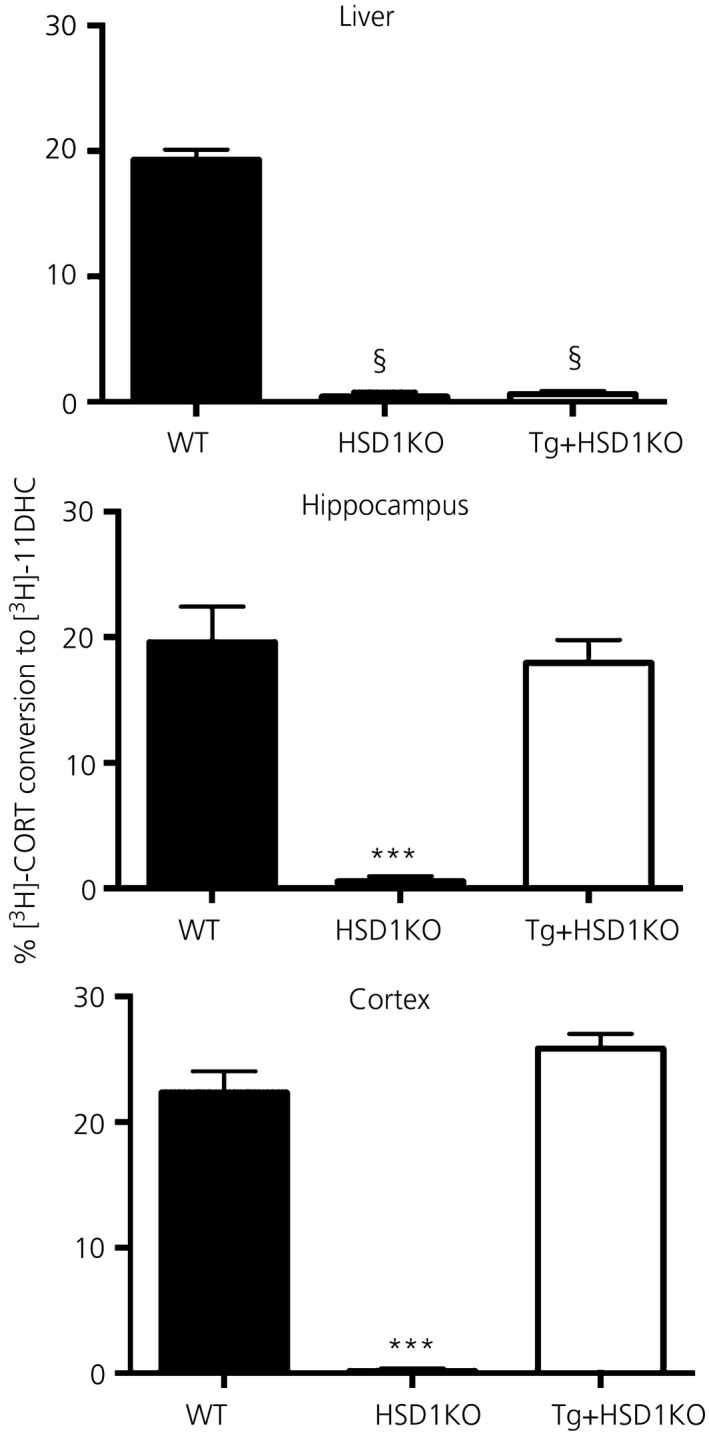
Effect of forebrain‐specific ‘rescue’ of 11β‐hydroxysteroid dehydrogenase type 1 (11β‐HSD1) in aged *hsd11b1* ^*−/−*^ mice on 11β‐HSD1 activity in brain and liver. 11β‐HSD1 enzyme activity measured *ex‐vivo* as percentage of [^3^H]‐corticosterone (CORT) conversion to [^3^H]‐11‐dehydrocorticosterone (11‐DHC) in hippocampus, cortex and liver homogenates from aged 24‐month‐old wild type (WT), *hsd11b1* ^*−/−*^ (HSD1KO) and Tg+HSD1KO mice (HSD1KO mice with 11β‐HSD1 rescued in the forebrain). §P < 0.0001 compared to WT; ***P < 0.0001 compared to WT and Tg+HSD1KO (n = 10–14 per genotype). Data are the mean ± SEM.

11β‐HSD1 activity (measured as 11β‐dehydrogenase conversion of [^3^H]‐CORT to [^3^H]‐11DHC in tissue homogenates) was present in the liver, hippocampus and cortex of WT controls but absent from all tissues tested in HSD1KO mice (Fig. 1). When HSD1KO mice were bred with transgenic mice with forebrain‐specific overexpression of 11β‐HSD1 (Tg mice) to generate Tg+HSD1KO mice, 11β‐HSD1 activity was recovered in the hippocampus and cortex to levels similar to WT controls but remained absent in liver (Fig. 1).

### Improved Y‐maze spatial memory in aged 11β‐HSD1 deficient mice reversed by CamIIK‐HSD1 transgene rescue

**Figure 2:**
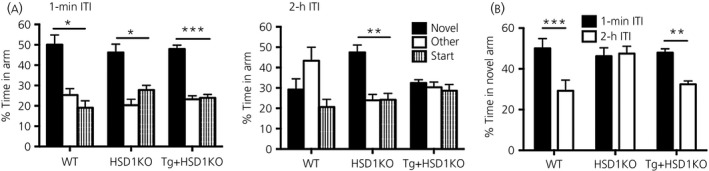
Forebrain specific expression of 11β‐hydroxysteroid dehydrogenase type 1 (11β‐HSD1) in aged *hsd11b1* ^*−/−*^ mice impairs spatial memory in the Y‐maze. (a) Data show within animal comparisons of percentage time spent in the novel, other and start arms following a 1‐min and 2‐h inter‐trial interval (ITI) for 24‐month‐old aged wild type (WT), *hsd11b1* ^*−/−*^ (HSD1KO) and Tg+HSD1KO mice. *P < 0.02, **P < 0.01, ***P < 0.0001 compared to the other two arms. (b) Comparison of percentage time spent in novel arm during the 1‐min and 2‐h ITI between genotypes. Spatial memory significantly impaired in WT and Tg+HSD1KO mice (*post‐hoc* Bonferroni's multiple comparisons test adjusted P‐values: ***P = 0.0006, **P = 0.0012 compared to corresponding 2‐h ITI) (n = 8–13 per genotype). Data are the mean ± SEM.

All groups of aged mice, WT, HSD1KO and Tg+HSD1KO, responded to novelty in the Y‐maze with no effect of genotype, spending significantly more time in novel arm than previously visited arms (P < 0.02) after a 1‐min ITI (Fig. 2 a). The percenatage time spent in the novel arm after a 2‐h ITI was significantly different from a 1‐min ITI (F_1,54_ = 18, P < 0.0001) with a genotype × time interaction effect (F_2,54_ = 5.4, P < 0.01) (Fig. 2 b). Aged HSD1KO mice spent more time in the novel arm (P < 0.05) than WT and Tg+HSD1KO mice after a 2‐h ITI, confirming the improved cognitive phenotype as described previously 13. Both aged WT and Tg+HSD1KO mice showed impaired spatial memory, spending less time in the novel arm after the 2‐h ITI than the 1‐min ITI (P < 0.01) (Fig. 2 b) and no more time in the novel arm than the previously visited arms (Fig. 2 a).

### Improved watermaze spatial learning in aged 11β‐HSD1‐deficient mice attenuated by CamIIK‐HSD1 transgene rescue

**Figure 3:**
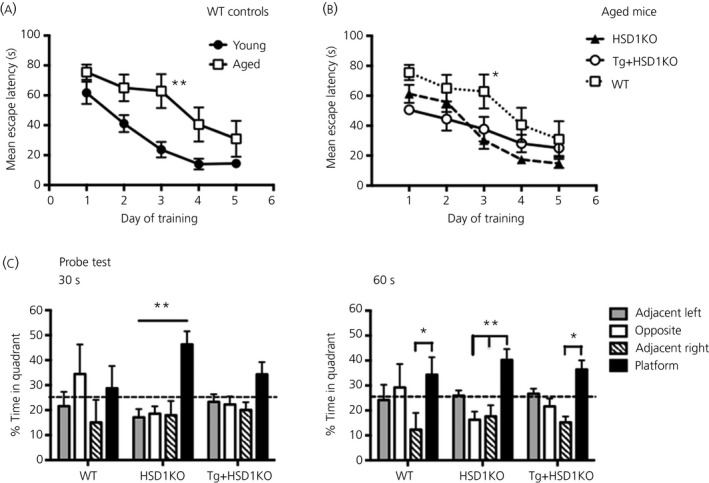
Effect of forebrain specific ‘rescue’ of 11β‐hydroxysteroid dehydrogenase type 1 (11β‐HSD1) in aged *hsd11b1* ^*−/−*^ mice on spatial learning in the watermaze. (a) Impaired spatial learning in aged (24‐month‐old) wild type (WT) mice (versus young (6‐month‐old) WT mice). **P < 0.01 compared to corresponding time point of young WT mice. (b) Spatial learning of aged (24‐month‐old) wild type (WT), *hsd11b1* ^*−/−*^ (HSD1KO) and Tg+HSD1KO mice. *P < 0.05 compared to corresponding time point of aged HSD1KO mice. (c) Spatial memory retention (24 h after last training trial) of aged (24‐month‐old) wild type (WT), *hsd11b1* ^*−/−*^ (HSD1KO) and Tg+HSD1KO mice. The percentage time in target quadrant was not significantly affected by genotype but within the groups there was an effect of quadrant (*post‐hoc* Bonferroni's multiple comparisons test adjusted P‐values: *P = 0.01, **P < 0.006 compared to the different quadrants of the pool as indicated) (n = 6–7 per genotype). Data are the mean ± SEM.

WT mice showed a decline in escape latencies over the 5 days of training trials (F_4,40_ = 15.63, P < 0.0001) (Fig. 3 a) indicating that learning but aged mice displayed longer escape latencies to find the hidden platform compared to young controls (age effect, F_1,10_ = 10.28, P < 0.01) (Fig. 3 a), thus confirming impaired spatial learning. Although aged WT, HSD1KO and Tg+HSD1KO mice all showed a decrease in escape latency effect over days of training (two‐way repeated measures anova, F_4,68_ = 27.62, P < 0.0001) (Fig. 3 b), there was a significant genotype effect (F_2,17_ = 3.9, P < 0.05) (Fig. 3 b). Aged HSD1 KO mice showed improved spatial learning (reduced escape latencies) compared to WT controls (F_1,11_ = 5.8, P < 0.05, Fig. 3 b). Spatial learning in Tg+HSD1KO mice, however, did not differ significantly from HSD1KO or WT mice, although there was a trend for better learning versus WT mice (F_1,11_ = 4.5, P = 0.06) (Fig. 3 b). These differences were not a consequence of vision or motor deficits in the aged mice because average daily swim speeds across the days of training did not differ significantly between genotypes and all mice were able to find the visible platform equally after 3 days of training (see Supporting information, Fig. S1). Interestingly, the escape latency on the first day of visible platform training was significantly lower in aged HSD1KO mice compared to both WT and Tg+HSD1KO mice. This quicker escape latency appears to reflect brain 11β‐HSD1 deficiency because this was not observed in the Tg+HSD1KO mice. However, any impact on overall performance of the HSD1KO mice is not clear because all groups learnt the visible platform position equally on the other 2 days.

Aged WT mice showed impaired spatial memory (24 h after the last training trial) during the probe trial (minus platform), failing to spend more time in the platform quadrant of the pool than other quadrants (Fig. 3 c). Aged HSD1KO but not Tg+HSD1KO mice displayed a preference for the target platform quadrant (F_3,68_ = 5.9, P = 0.001) (Fig. 3 c) during the first 30 s of the probe trial, indicating intact spatial memory of the platform location, although the percenatage time in the target quadrant was not significantly greater than in aged WT mice. When the full 60 s of the probe test was analysed, aged HSD1KO mice no longer showed a significant preference for the target quadrant over all the other quadrants (Fig. 3 c). This is consistent with a decrease in target quadrant preference after the first 30 s 27.

### Increased BDNF mRNA levels in the hippocampus of aged 11β‐HSD1‐deficient but not Tg+HSD1 KO mice

**Figure 4:**
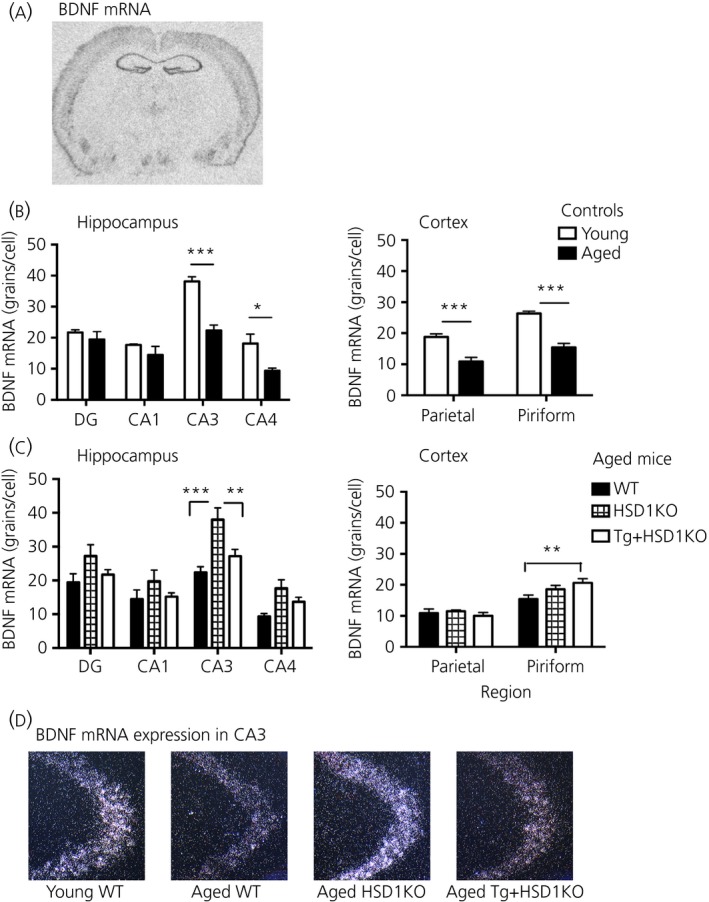
Effect of forebrain‐specific ‘rescue’ of 11β‐hydroxysteroid dehydrogenase type 1 (11β‐HSD1) in aged *hsd11b1* ^*−/−*^ mice on brain‐derived neurotrophic factor (BDNF) mRNA levels in the hippocampus and cortex. (a) Autoradiograph showing distribution of BDNF mRNA in coronal section at the level of the dorsal hippocampus from a control adult C57BL6/J brain. (b) BDNF mRNA levels in hippocampal subregions and cortex of young 6‐month‐old and aged 24‐month‐old C57BL6/J control mice. (c) BDNF mRNA levels in hippocampal subregions and cortex of aged 24‐month‐old wild type (WT), *hsd11b1* ^*−/−*^ (HSD1KO) and Tg+HSD1KO mice (n = 6–7 per genotype). Data are the mean ± SEM. (d) Representative dark‐field photomicrographs of *in situ* hybridisation showing BDNF mRNA in the CA3 subregion of the hippocampus from wild type (WT; young 6‐month‐old and aged 24‐month‐old) aged HSD1KO and aged Tg+HSD1KO mice. Autoradiographic silver grains appear white. Post‐hoc Bonferroni's multiple comparisons test adjusted P‐values: ***P < 0.0001, **P < 0.008, *P < 0.03 compared to the corresponding aged mice as indicated.

BDNF mRNA was highly expressed in all hippocampal regions, particularly CA3 and in the cortex of control WT mice (Fig. 4 a,b). Compared to young WT mice, BDNF mRNA levels in aged WT mice were significantly decreased with age in hippocampal CA3 and CA4 (approximately 41% and 48% decrease, respectively; F_1,32_ = 25.88, P < 0.0001) (Fig. 4 b) and in the parietal and piriform cortex (approximately 42% and 39% decrease, respectively; F_1,16_ = 54.9, P < 0.0001) (Fig. 4 b). In the aged (24 months) WT, HSD1KO and TG+HSD1KO mice, BDNF mRNA expression was significantly altered by genotype (F_2,68_ = 15.5, P < 0.0001) and hippocampal region (F_3,68_ = 25.13, P < 0.0001) (Fig. 4 c). BDNF mRNA levels were increased selectively in CA3 hippocampal cells (approximately 70% increase, P < 0.001) in aged HSD1KO mice compared to WT mice (Fig. 4 c,d). Rescue of 11β‐HSD1 expression in the forebrain reduced BDNF mRNA in CA3 of Tg+HSD1KO mice (P < 0.05, compared to HSD1KO mice) to levels not different from aged WT controls (Fig. 4 c,d). BDNF mRNA expression was not affected by genotype in the cortex, although there was a significant genotype × region interaction (F_2,34_ = 3.54, P < 0.05) (Fig. 4 c). Tg+HSD1KO mice showed a small increase in BDNF mRNA levels compared to WT controls in the piriform cortex (Fig. 4 c). Hippocampal MR and GR mRNA levels, in contrast, were not altered in either aged HSD1KO or Tg+HSD1KO mice (see Supporting information, Fig. S2).

### Hyperplasia of adrenal glands in aged 11β‐HSD1 deficient mice not rescued by CamIIK‐HSD1 transgene

The left adrenals were significantly enlarged by approximately 30% (F_2,31_ = 4.7, P < 0.05) in aged HSD1KO mice compared to aged WT controls (see Supporting information, Table S2). Aged 11β‐HSD1‐deficient mice with the CamIIK‐HSD1 transgene rescue of 11β‐HSD1 activity in the forebrain (Tg+HSD1KO) showed a nonsignificant trend for heavier adrenals (see Supporting information, Table S2). Total adrenal weights (left and right) when expressed relative to body weight were significantly increased by approximately 33% (F_2,30_ = 4.67, P < 0.05) in both aged HSD1KO and Tg+HSD1KO mice compared to WT mice (see Supporting information, Table S2). Basal plasma CORT levels were not significantly different between aged HSD1KO, Tg+HSD1KO and WT mice (see Supporting information, Table S2).

## Discussion

The main finding from the present study is that CamIIK‐HSD1 transgene rescue of 11β‐HSD1 activity in the forebrain reversed both increased hippocampal BDNF mRNA and improved spatial memory in aged 11β‐HSD1 deficient mice to aged WT mice levels. This implicates 11β‐HSD1 activity in the forebrain as a key contributor to the mechanisms that underlie impaired spatial memory in aged WT mice. Furthermore, 11β‐HSD1 activity outside the brain appears to make some contribution towards the cognitive effects of aged mice, at least under stress conditions, such as in the watermaze.

11β‐HSD1‐deficient mice have been shown to resist hyperglycaemia provoked by obesity or stress reflecting reduced intracellular GC levels in adipose and liver 14, 23. Because hyperglycaemia and hyperinsulinaemia associate with cognitive decline in humans and some animal models 16, 28, 29, 30, a deficiency of 11β‐HSD1 in liver and adipose tissues could potentially contribute to the cognitive phenotype of aged 11β‐HSD1‐deficient mice. We previously reported no change in glucose tolerance but increased insulin sensitivity in aged 11β‐HSD1‐deficient mice, suggesting a possible influence on cognitive function 13. Aged 11β‐HSD1 deficient mice with CamIIK‐HSD1 transgene rescue of 11β‐HSD1 activity in forebrain no longer showed enhanced spatial memory in the Y‐maze with a similar performance to aged WT mice. These data suggest that peripheral 11β‐HSD1 activity has little effect on spatial memory in aged mice under basal conditions in the Y‐maze. However, when tested in the more stressful watermaze task, the improved spatial learning in aged 11β‐HSD1 deficient mice 13 was not fully reversed with CamIIK‐HSD1 transgene rescue of 11β‐HSD1 in the forebrain. Instead, aged Tg+HSD1KO mice show spatial learning performances that were between aged WT mice and aged 11β‐HSD1 deficient mice with no significant difference between the genotypes. Hyperglycaemia and increased CORT levels associated with stress impairs hippocampal neurogenesis, synaptic plasticity and learning 31, whereas 11β‐HSD1‐deficient mice have been shown to resist hyperglycaemia induced by stress or obesity 14, 15. Thus, the beneficial cognitive effects of peripheral deficiency of 11β‐HSD1 in the aged Tg+HSD1KO mice may only become apparent under conditions of stress, such as in the watermaze.

Adrenal weights are increased in aged 11β‐HSD1‐deficient mice compared to aged WT controls 13 as would be anticipated for increased CORT production to compensate for reduced local production at brain feedback sites 22. In the present study, the increased adrenal size was not associated with increased basal plasma CORT levels as observed in 11β‐HSD1‐deficient mice on the 129 strain 22, 24, 32, thus confirming a lack of effect on the C57BL/6J strain 13. CamIIK‐HSD1 transgene rescue of 11β‐HSD1 activity in the forebrain, however, did not reverse the increased adrenal size relative to body weight in aged 11β‐HSD1‐deficient mice. This may reflect reduced bulk CORT synthesis as a result of a lack of 11β‐HSD1 in tissues outisde the forebrain, notably the liver of aged Tg+HSD1KO mice. Indeed, transgene rescue of 11β‐HSD1 activity in liver restores hyperplasia of the adrenal gland in 11β‐HSD1‐deficient mice, supporting a major influence of hepatic GC metabolism on HPA function 33.

BDNF is well established to play an essential role as a mediator of neural plasticity and consequently memory formation, particularly in hippocampus‐dependent tasks 34, 35. BDNF levels in the hippocampus have been shown to decline with age in rats 36, 37, as well as in humans 38. Our observed decrease in BDNF mRNA expression in the hippocampus of aged WT mice relative to young controls may contribute to their impaired spatial memory. In support, BDNF mRNA expression in the hippocampus of non‐impaired and impaired aged rats correlates with their spatial memory performance in the watermaze 39. Moreover, chronic deficiency of BDNF in mice leads to impaired spatial memory in an age‐dependent manner 40. Several studies have also shown increases in hippocampal BDNF mRNA expression following a learning task in animals with improved memory but no change in BDNF mRNA levels in those with impaired memory 39, 41, 42. In line with these studies, the increased BDNF mRNA in the CA3 sub‐region of the hippocampus in aged 11β‐HSD1‐deficient mice was associated with improved spatial memory, whereas aged WT mice with lower BDNF mRNA were impaired in both Y‐maze and watermaze. Although hippocampal BDNF mRNA levels in aged 11β‐HSD1‐deficient mice were similar to young WT mice, a lack of effect of ageing remains to be confirmed with the inclusion of young 11β‐HSD1‐deficient mice.

The regulation of the BDNF gene is complex, with at least eight differentially regulated promoters each giving rise to mRNA transcripts with a distinct 5′ exon spliced to a common 3′ coding exon but with all transcripts encoding an identical BDNF protein 43. As with most studies, we investigated the effect of age on total BDNF mRNA in the WT mice so that any change in one or more specific BDNF mRNA splice transcripts is not known. However, the decrease in total BDNF mRNA in the CA3 hippocampus in the aged WT mice may reflect a decrease in all the four predominant 5′ exon‐specific transcripts with age 37.

The neuronal‐specific transcription factor NPAS4 is a key regulator of brain plasticity and cognitive function 44. We have previously found decreased Npas4 mRNA in the hippocampus of aged memory impaired WT mice but maintained levels in aged 11β‐HSD1‐deficient mice 45. Because BDNF is one of the target genes of Npas4, the reduced BDNF mRNA in the hippocampus of aged WT mice may be a consequence of reduced Npas4 levels. Both Npas4 and BDNF mRNA in the hippocampus have been shown to decrease with age in rats 46. Moreover, both stress and elevated levels of CORT down regulate hippocampal BDNF mRNA expression 47, 48, 49. Thus, the lower BDNF mRNA levels in CA3 hippocampus of aged WT and aged Tg+HSD1KO mice compared to aged 11β‐HSD1‐deficient mice may be the result of increased intracellular CORT levels by 11β‐HSD1 activity in the brain.

In conclusion, the rescue of 11β‐HSD1 in the forebrain of global 11β‐HSD1‐deficient mice appears to be sufficient to re‐instate the age‐associated spatial memory deficits in the Y‐maze, an effect that is associated with lower BDNF but not MR or GR mRNA expression in the hippocampus. Interestingly, the improved spatial learning in the watermaze was not fully reversed in the aged Tg+HSD1KO mice, suggesting a contribution of peripheral 11β‐HSD1 deficiency to cognitive function under stress conditions, such as during the watermaze task.

## Supporting information


**Table S1.** Oligonucleotide sequences for polymerase chain reaction amplification of ‘floppy’ cDNA template for brain‐derived neurotrophic factor (BDNF) probe synthesis.
**Table S2.** Adrenal weight, body weight and basal AM plasma corticosterone (CORT) levels in aged behaviourally tested aged mice.
**Fig. S1.** 11β‐Hydroxysteroid dehydrogenase type 1 (11β‐HSD1) deficiency and forebrain specific transgene rescue of 11β‐HSD1 in aged 11β‐HSD1 deficient mice has no effect on swim speeds or vision in the watermaze.
**Fig. S2.** 11β‐Hydroxysteroid dehydrogenase type 1 (11β‐HSD1) deficiency and forebrain specific transgene rescue of 11β‐HSD1 in aged 11β‐HSD1 deficient mice do not alter hippocampal mineralocorticoid receptor (MR) and glucocorticoid receptor (GR) mRNA expression.
