# Supplementary material for: Forebrain‐Specific Transgene Rescue of 11β‐HSD1 Associates with Impaired Spatial Memory and Reduced Hippocampal Brain‐Derived Neurotrophic Factor mRNA Levels in Aged 11β‐HSD1 Deficient Mice
Source: J Neuroendocrinol. 2017 Jan 9;29(1):10.1111/jne.12447. doi: 10.1111/jne.12447 (PMC5244685; doi:10.1111/jne.12447)
Supplement: Supplementary file 1 — Table S1. Oligonucleotide sequences for polymerase chain reaction amplification of ‘floppy’ cDNA template for brain‐derived neurotrophic factor (BDNF) probe synthesis. Table S2. Adrenal weight, body weight and basal AM plasma corticosterone (CORT) levels in aged behaviourally tested aged mice. Fig. S1. 11β‐Hydroxysteroid dehydrogenase type 1 (11β‐HSD1) deficiency and forebrain specific transgene rescue of 11β‐HSD1 in aged 11β‐HSD1 deficient mice has no effect on swim speeds or vision in the watermaze. Fig. S2. 11β‐Hydroxysteroid dehydrogenase type 1 (11β‐HSD1) deficiency and forebrain specific transgene rescue of 11β‐HSD1 in aged 11β‐HSD1 deficient mice do not alter hippocampal mineralocorticoid receptor (MR) and glucocorticoid receptor (GR) mRNA expression. [file JUNE-29-12447-s001.pdf]

Supplementary information

Forebrain specific transgene rescue of 11β-HSD1 associates with impaired spatial memory and reduced hippocampal BDNF mRNA levels in aged 11β-HSD1 deficient mice

Sarah Caughey[1, 2], Anjanette P. Harris[1, 2], Jonathan R. Seckl[1, 2], Megan C. Holmes[1, 2] and Joyce L.W. Yau[1, 2]

[1]BHF Centre for Cardiovascular Science and [2]Centre for Cognitive Ageing and Cognitive Epidemiology, University of Edinburgh, UK

Corresponding author:
Dr Joyce LW Yau**,**
BHF Centre for Cardiovascular Science,
Queen's Medical Research Institute,
47 Little France Crescent,
Edinburgh EH16 4TJ
UK
Tel: +44 (0) 131 242 6760
Fax: +44 (0) 131 242 6779
E-mail: Joyce.Yau@ed.ac.uk

Table S1. Oligonucleotide sequences for PCR amplification of 'floppy' cDNA template for BDNF probe synthesis

	Primer sequences (non highlighted sequence are linker bases)
Forward (sense)	5'-GATAATACGACTCACTATATAGGG GAGATGGCTGACACTTTTGAGC-3'
Reverse (antisense)	5'- GAATTAACCCTCACTAAAGGGAGAGACTTATGAATCGCCTGCC-3'

T7 RNA polymerase sequence highlighted in blue; T3 RNA polymerase sequence in pink; BDNF sequence in yellow

Table S2. Adrenal weight, body weight and basal AM plasma CORT levels in aged behaviourally tested aged mice

	Body weight (g)	Adrenal weights (mg)		Total adrenal/body weight (mg/g)	Plasma CORT (nM)
		Left	Right		
WT	40.6 ± 2.6	1.78 ± 0.14	1.65 ± 0.23	0.09 ± 0.01	40.6 ± 2.6
HSD1KO	38 ± 2.1	2.35 ± 0.15*	1.98 ± 0.07	0.12 ± 0.01*	38.1 ± 2.1
Tg+HSD1KO	36.2 ± 1.6	2.12 ± 0.09	2.04 ± 0.18	0.12 ± 0.01*	36.2 ± 1.6

*P<0.05 vs WT. Data are expressed as mean ± sem, n=9-14/genotype.

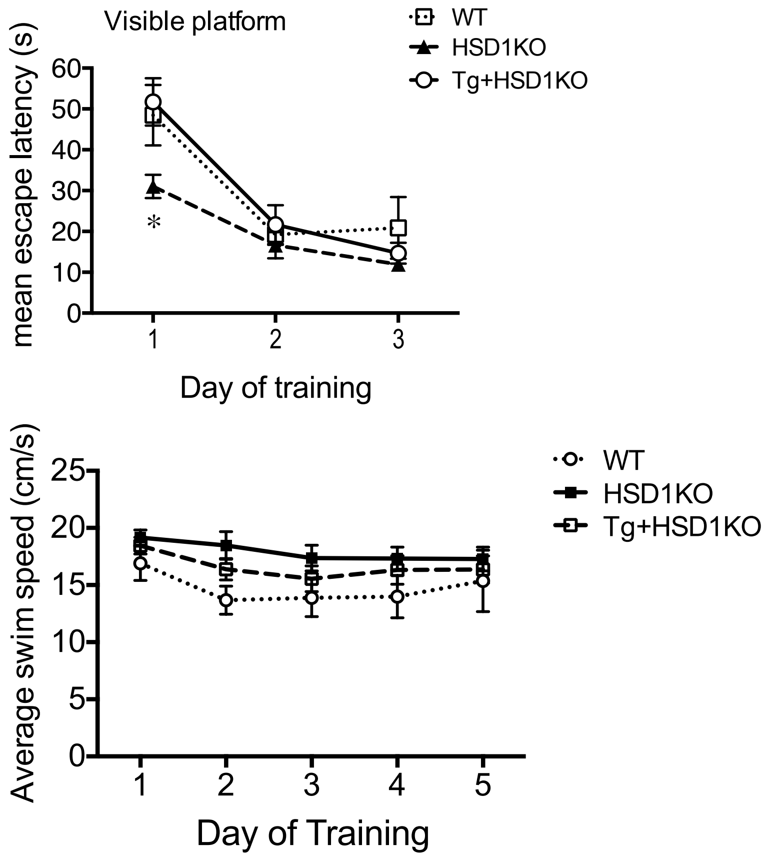

Figure S1. 11β-HSD1 deficiency and forebrain specific transgene rescue of 11β-HSD1 in aged 11β-HSD1 deficient mice has no effect on swim speeds or vision in the watermaze. Performance of mice in the visible platform version of the watermaze and average swim speeds over the days of training in the hidden platform task are shown for 24 months-old wild type (WT), *hsd11b1*$^{-/-}$ (HSD1KO), and Tg+HSD1KO mice. Data were analysed with two-way repeated measures ANOVA. Significance was determined by *post-hoc* Bonferroni's multiple comparisons test, adjusted P value: * P= 0.03 compared to WT and P=0.005 compared to Tg+HSD1KO. Data are means ± SEM.

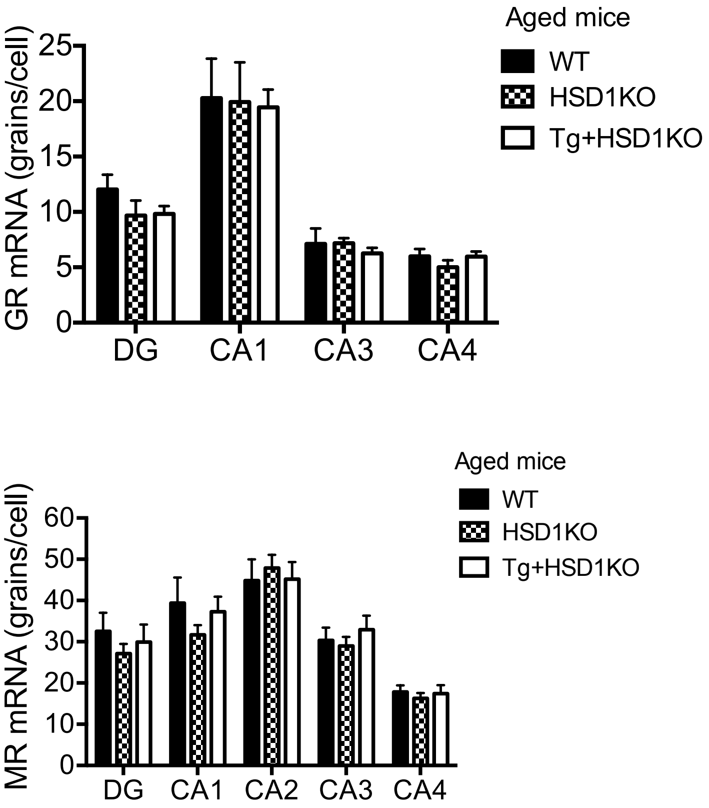

Figure S2. 11β-HSD1 deficiency and forebrain specific transgene rescue of 11β-HSD1 in aged 11β-HSD1 deficient mice do not alter hippocampal MR and GR mRNA expression. MR and GR mRNA levels are expressed as autographic (photographic emulsion) silver grains per cell area in the hippocampus dentate gyrus *DG) and CA sub-regions of aged 24 month-old wild type (WT), *hsd11b1*$^{-/-}$ (HSD1KO), and Tg+HSD1KO mice. Data are means ± SEM.
